# Complication Profile and Safety Outcomes of Aquablation in the Management of BPH

**DOI:** 10.3390/medicina61122076

**Published:** 2025-11-21

**Authors:** Panagiotis Triantafyllou, Polyvios Arseniou, Stamatios Katsimperis, Ioannis Kyriazis, Ioannis Manolitsis, Patrick Juliebø-Jones, Bhaskar Somani, Arman Tsaturyan, Theodoros Karagiotis, Titos Markopoulos, Lazaros Tzelves, Andreas Skolarikos

**Affiliations:** 1 Second Department of Urology, National and Kapodistrian University of Athens, Sismanogleio General Hospital of Attica, 15126 Marousi, Greece; 2 European Association of Urology-Young Academic Urologists (EAU-YAU), Urolithiasis and Endourology Working Group, 6842 CVArnhem, The Netherlands; 3 Department of Urology, Aberdeen Royal Infirmary, NHS Grampian, Aberdeen AB25 2ZN, UK; 4 Department of Urology, Haukeland University Hospital, 5009 Bergen, Norway; 5 Department of Urology, University Hospital Southampton NHS Foundation Trust, Southampton SO16 6YD, UK; 6 Department of Urology, Yerevan State Medical University After Mkhitar Heratsi, Yerevan 0025, Armenia

**Keywords:** Aquablation, benign prostatic hyperplasia, minimally invasive surgery, complications, safety

## Abstract

*Background and Objectives*: Aquablation, a robot-assisted, heat-free resection technique, has emerged as a promising minimally invasive surgical therapy (MIST) for benign prostatic hyperplasia (BPH). Its precision and potential for preservation of sexual function distinguish it from traditional surgical options. This systematic review aimed to evaluate the safety profile of Aquablation, with emphasis on perioperative complications and functional outcomes. *Materials and Methods*: We systematically searched PubMed/MEDLINE, Scopus, and the Cochrane Library through June 2025 in accordance with PRISMA 2020 guidelines (PROSPERO CRD420251074479). Eligible studies included randomized and non-randomized trials of Aquablation in men with BPH, reporting adverse events by type, frequency, or severity. Risk of bias was assessed with ROB-2 and ROBINS-I tools. *Results*: Forty-seven studies were included, spanning randomized controlled and prospective observational designs. Most complications were minor (Clavien-Dindo grade 1–2). Bleeding was the most frequent perioperative event, with transfusion required in 1–8% of cases, more often in large prostates. Severe complications (grade ≥ 3) were uncommon and usually related to bleeding or urinary retention. Long-term sequelae such as strictures or bladder neck contracture were rare. Compared with transurethral resection of the prostate, Aquablation yielded lower rates of ejaculatory dysfunction (10% vs. 36%), with continence and erectile function largely preserved. Outcomes were comparable to holmium laser enucleation, but Aquablation demonstrated superior ejaculatory preservation. *Conclusions*: Aquablation demonstrates a favorable safety profile across prostate sizes, with its greatest advantage being preservation of sexual function. While bleeding remains the principal perioperative concern, life-threatening events are rare. Further independent, long-term comparative studies are warranted.

## 1. Introduction

Benign prostatic hyperplasia (BPH) is a common condition among aging men and its associated lower urinary tract symptoms (LUTS) often lead to a significant decline in quality of life [1]. For decades, transurethral resection of the prostate (TURP) has been regarded as the gold standard of surgical treatment for men with moderate to severe symptoms refractory to medical therapy [1]. However, the perioperative risks and long-term side effects associated with TURP, including retrograde ejaculation and erectile dysfunction, have prompted the development of alternatives that aim to achieve similar efficacy while reducing morbidity [1,2]. Over the past two decades, minimally invasive surgical therapies (MISTs) have emerged as valuable options to address this clinical need. These therapies include holmium and thulium laser enucleation techniques (HoLEP, ThuLEP), photoselective vaporization of the prostate (PVP), convective water vapor thermal therapy (Rezūm/WVTT), prostatic artery embolization (PAE), transurethral heat- or radiofrequency-based approaches, such as TUNA and TUMT, prostatic urethral lift (UroLift/PUL), temporary implantable nitinol device (iTind) and Optilume, all of which are designed to improve urinary flow and relieve obstruction with a shorter learning curve, reduced operative time, and enhanced preservation of functional outcomes compared with traditional resection methods [1,2,3].

Aquablation has recently been introduced as a novel addition to the family of MISTs [4]. This technique combines real-time transrectal ultrasound imaging with cystoscopic guidance to generate a detailed surgical map of the prostate [3]. Once the operative plan is established, a robotically controlled high-velocity saline waterjet is employed to resect the targeted tissue in a heat-free manner [4]. The surgeon remains in control of the procedure, while the robotic platform ensures precision in executing the predetermined contours of tissue removal [3]. This integration of imaging, surgical planning, and robotic execution allows Aquablation to provide consistent resection across a wide spectrum of prostate sizes and shapes, a feature that distinguishes it from other minimally invasive approaches [1,4].

The absence of thermal energy during Aquablation is considered an important characteristic of the technique, as it is intended to minimize collateral damage to surrounding structures critical for urinary and sexual function [4]. Furthermore, the use of robotic assistance reduces variability between operators and enhances procedural reproducibility [3]. These attributes have positioned Aquablation as a promising alternative for men with symptomatic BPH, and its adoption has expanded rapidly in recent years [1], typically for prostates under 80 mL and for men that wish to maintain the ability to ejaculate [2]. Multiple clinical studies have now investigated its role relative to TURP and other minimally invasive techniques, highlighting the need for a focused evaluation of outcomes associated with this therapy [2,4]. Given its increasing adoption and distinct technical profile, it is consequently essential to examine the complications associated with Aquablation [1,3].

## 2. Materials and Methods

### 2.1. Protocol and Registration

This systematic review was prospectively registered in PROSPERO (CRD420251074479). The review was designed and reported in accordance with the Preferred Reporting Items for Systematic Reviews and Meta-Analyses (PRISMA) 2020 statement.

### 2.2. Eligibility Criteria

We included studies that investigated adult male patients with benign prostatic hyperplasia undergoing Aquablation as a minimally invasive surgical therapy. Both randomized and non-randomized study designs were eligible, provided they reported complications related to Aquablation. Studies were required to provide sufficient detail regarding the type, frequency, or severity of complications, with classification according to the Clavien-Dindo (CD) grading system recorded where available. No comparator arm was required for inclusion. Only articles published in English were considered, with no restrictions on publication date. Review articles, editorials, notes and letters, guidelines, protocols, articles without full-text data, and animal studies were excluded.

### 2.3. Search Strategy

A comprehensive search was conducted in PubMed/MEDLINE, Scopus and The Cochrane Library (CLIB). Only published studies were sought. The search strategy combined free-text form of the terms Aquablation and complications. The last search was conducted on 22 June 2025.

### 2.4. Study Selection and Data Extraction

**Figure 1:**
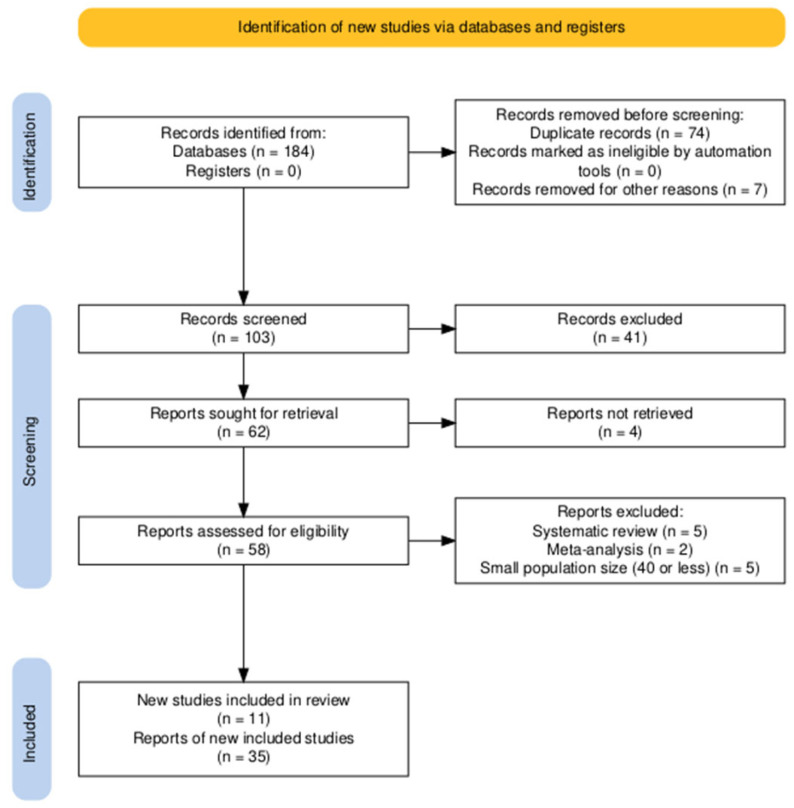
PRISMA 2020 flow diagram.

All retrieved records were imported into Zotero (Version 7.0.16, Digital Scholar, Vienna, VA, USA) for reference management, and duplicates were removed. Two reviewers (P.T. and P.A.) independently screened titles and abstracts to assess potential eligibility, followed by full-text review of selected articles. Disagreements at any stage were resolved through discussion with a third and fourth reviewer (S.K. and I.K.) until consensus was reached. The PRISMA 2020 flow diagram of the study selection process is presented in Figure 1 [5]. is A standardized spreadsheet created in Microsoft Excel was used to maintain a data log of screening decisions and extracted data. For each included study, two reviewers (P.T. and P.A.) independently extracted study characteristics, intervention details, and all reported complications of Aquablation. Extracted data were cross-checked for accuracy (S.K.), and discrepancies were resolved by consensus, during meetings (with S.K. and I.K.).

### 2.5. Risk of Bias Assessment

The risk of bias in randomized controlled studies was assessed using the ROB-2 tool. For non-randomized studies, the risk of bias was assessed using the ROBINS-I tool. Two reviewers (P.T. and P.A.) independently performed the assessment, and disagreements were resolved by consensus (with the guidance of T.K. and T.M.).

### 2.6. Data Synthesis

Given the heterogeneity of the available studies and the descriptive nature of the review objective, no formal meta-analysis was planned. Findings were synthesized narratively and presented in tabular form, with complications summarized by type, frequency, and severity. Where reported, grading of complications according to the Clavien-Dindo classification was included.

## 3. Results

One of the first studies that investigated the safety profile of Aquablation was the WATER trial, a double-blind, multi-center, randomized clinical trial, which included the comparison of Aquablation to TURP [6]. In this trial, the overall incidence of the primary safety point was significantly lower in the Aquablation group compared to TURP (26% vs. 42%, *p* = 0.0149). Persistent grade 1 events, including ejaculatory dysfunction, erectile dysfunction, and incontinence, occurred in 7% of patients of the Aquablation group and 25% following TURP (*p* = 0.0004). Most common grade 1 event was bleeding and dysuria in the Aquablation arm and retro-ejaculation in the TURP arm. The rate of grade ≥2 adverse events did not differ significantly between groups (20% vs. 23%, *p* = 0.30). Anejaculation was observed less frequently with Aquablation than with TURP (10% vs. 36%, *p* = 0.0003), making this a good advantage of this surgical technique. In the subgroup with prostate volumes ≥ 50 mL, the difference was even more pronounced (2% vs. 41%, *p* = 0.0001).

Additionally, other specific perioperative complications were uncommon. Only one Aquablation patient required transfusion, whereas no transfusions were reported in the TURP group. Urethral strictures or adhesions occurred in 3 Aquablation patients and 2 TURP patients (CD grade 3a/3b). Urinary retention was reported in 9 patients after Aquablation and 4 after TURP (grade 1), with one additional grade 3b retention event in the Aquablation arm. Urinary tract infection (UTI) cases were 9 vs. 5 (Aquablation vs. TURP, grade 2). A single case of arrhythmia (grade 4) occurred following Aquablation, with none in the TURP group.

Across all 5 years of follow-up, changes in ejaculatory function remained minimal in the Aquablation arm, while TURP patients reported sustained worsening, with an average 2.7-point decline in MSHQ-EjD scores compared to Aquablation (*p* = 0.0015) [7]. From 3 to 5 years, there were no significant differences in urinary complication rates between the groups [7,8].

Desai et al. in 2019 reported the procedural outcomes of the WATER II, a multi-center prospective clinical trial [9]. A number of 101 patients with prostates 80–150 mL underwent Aquablation. At one month, 112 adverse events were reported in 55 patients (54.5%) where the rate of CD grade ≥ 2 events was 29.7%. Bleeding-related complications represented the most commonly observed adverse events. In-hospital hematuria events occurred in 10 patients (9.9%), including 6 perioperative transfusions (5.9%). Additional delayed hemorrhage requiring transfusion or cystoscopic fulguration was observed in 6 patients. Overall, 8 patients (7.9%) required transfusion and/or endoscopic clot evacuation or fulguration. When summarized, the distributions of grade 1, 2, 3 and 4 events were 30.7%, 18.8%, 10.9% and 5%, respectively. One sexual adverse event reported in one patient (unspecified). During the 12-month follow-up, among the sexually active men, 81% maintained antegrade ejaculation, there were no late bleeding events and no need for re-operation. During the late follow-up period (12–36 months), complication rates remained low, with no new cases of major bleeding. Moreover, new erectile or ejaculatory dysfunction was observed in 2% of patients each, and persistent incontinence was reported at a very low rate of 2%. By the 5 years follow-up, no bladder neck contractures or urethral stenoses were reported. Long-term erectile and ejaculatory function was consistent with baseline measurements, and no instances of newly developed permanent dysfunction were observed.

In 2020, Bach et al. conducted the first multi-center, prospective trial with no prostate size limits [10]. During this trial, 178 patients were enrolled from 5 international centers. Most complications were minor (69 events over 56 subjects). There were 33 grade-1 and 15 grade-2 events reported. Transfusion was needed in 2.7% of cases, and 7.9% returned to surgery for clot evacuation or cautery, usually early after the operation. Sexual function was mostly maintained, only 8% experienced ejaculatory dysfunction, with minimal issues in erectile function or continence. Furthermore, CD grade 3b complications occurred in 9% of patients (16 events), mostly related to bleeding, with one case each of rectal perforation and urethral stricture.

Between 2019 and 2025, Omidele et al., in a prospective, single-center observational study that included a total of 330 patients with mean prostatic volume 110 mL, found the most frequent complication within the first month to be UTI (37 patients, 11.2%) [11]. Bleeding requiring transfusion occurred in 11 patients (3.3%). At 4 years, the overall surgical re-treatment rate was 3.9% (2 cases for post-operative bleeding, 1 case of bladder neck disruption, and 10 cases requiring TURP because of residual anterior prostatic tissue. Most importantly, antegrade ejaculation was preserved in 99.6% of men, forming Aquablation sexual safety profile. Throughout the follow-up period, there were no reports of new cases of long-term incontinence, urethral stricture, or bladder neck contracture.

In a single-center retrospective study which included 174 patients who underwent Aquablation from January 2021 to September 2022, Ringler et al. stratified the patients according to prostate volume: PV ≥ 150 (33 patients) versus PV < 150 (123 patients) [12]. Overall, 33 patients (19%) experienced an adverse event, with a mean CD grade of 1.76. Only four men experienced a grade ≥ 3 complication: one with grade 3, two with grade 4, and one with grade 5 event. Transfusion was needed in 2.3% of cases (4 patients), and the mean hemoglobin drop was 1.78 g/dL. The surgical re-treatment rate was 12.9% during a mean follow-up of 20–34 months. When categorized by prostate volume, men with PV ≥ 150 mL had higher re-treatment rates (24.2% vs. 10.7%, *p* = 0.043) and were more likely to remain on 5-ARIs post-operatively (31.3% vs. 8.9%, *p* = 0.04). Adverse events categorized by prostate size showed no significant differences in rates or severity (mean CD grade 1.71 vs. 1.67, *p* = 0.908). The ≥150 mL group had a greater hemoglobin decline (2.45 vs. 1.54 g/dL, *p* = 0.004), but transfusion rates did not differ significantly (3.0% vs. 1.6%, *p* = 0.607).

Quintas et al., in a prospective, multi-center comparative study including 150 men (75 Aquablation, 75 HoLEP), found no intraoperative complications reported in either group [13]. The mean hemoglobin drop was significantly greater in the Aquablation arm (2.6 g/dL vs. 0.4 g/dL, *p* < 0.001), although this did not translate into higher transfusion rates, which were identical at 1.3% in both groups. The rates of emergency visit (13.3% HoLEP vs. 10.6% Aquablation, *p* = 0.93), re-admission (2.7% vs. 4%, *p* = 0.39) and re-catheterization (9.3% vs. 5.3%, *p* = 0.75) within the first month were also similar. At 6 months, ejaculatory dysfunction was markedly more common following HoLEP (89.3%), compared to Aquablation (6.6%, *p* < 0.001). Furthermore, social continence was maintained in all Aquablation patients, while three HoLEP subjects reported incontinence at 6 months. Erectile function (IIEF-15 score calculation) showed no significant decline from baseline in either group.

During a prospective, multi-center Aquablation survey of 103 Japanese men with BPH, Hinata et al. reported a favorable 30-day safety profile. In detail, there were no cases of pad-use incontinence, erectile dysfunction, or ejaculatory dysfunction reported, and no device-related serious adverse events [14]. Only one patient experienced a CD grade 3 adverse event, requiring transfusion and fulguration for bleeding on post-operative day 11, which was attributed to straining during voiding in a man who had been catheter-dependent preoperatively. However, over 6 months, procedure-related complications were reported in 10.7% of patients, with UTI (2 cases) and urethral stricture (3 cases) being the most common. It is worth mentioning that no patient required a secondary BPH intervention.

Similarly, in 2018, Bach et al., between 118 men who underwent Aquablation, found a promising safety profile [15]. Specifically, 13 adverse events greater than grade 1 occurred in 10 patients (8.5%). Most events were grade 2, including re-catheterization for urinary retention and three cases (2.5%) requiring transfusion for clinically significant hematuria. Grade 3b events were reported in four patients (3.4%), each requiring secondary endoscopic electrocauterization. In addition, two patients were re-admitted within 2 weeks for non-severe complications (one with antibiotic-amenable UTI and one with hematuria without needing intervention). The mean hemoglobin decline was 1.8 g/dL (14.2 to 12.4 g/dL, *p* < 0.001). Electrocautery was used during surgery in only 3.4% of cases. There were no bladder neck contractures, urethral strictures, or de novo incontinence reported during the early 3-month follow-up. Most importantly, sexual function was preserved, with 73% of men retaining antegrade ejaculation. No patients required surgical re-treatment for recurrent LUTS within the follow-up period.

Several additional single-center clinical trials evaluating Aquablation have been reported in recent years and were incorporated into this systematic review. Labban et al., in a prospective study of 59 men, reported an adverse event rate of 22% (14 events in 13 patients) [16]. The most frequent events were grade 1–2 (71.4%), with acute urinary retention being the most common. Less common were grade 3a (reduced urinary flow required cystoscopy) and 3b events (urethral stricture). There was a single urosepsis event (grade 4a). Ejaculatory preservation was achieved in 82.8% at 3 months and 84.6% at 12 months. No patient developed newly acquired permanent incontinence. In addition, Kasraeian et al., in a 2020 prospective study of 55 men, observed that the majority of adverse events were minor [17]. Overall, 9 men presented complications which included hematuria (9%), bladder spasm (1.8%), dehydration (1.8%), foley catheter intolerance (1.8%) and a temporary rise in creatinine (1.8%). The ejaculatory function was not systematically reported.

Furthermore, Desai et al., in a single-center prospective trial with 47 patients from India, registered a total of 10 complications in 8 patients [18]. Namely, three cases of urinary retention (grade 1), one case of hematuria and one of UTI (grade 2) and five cases requiring re-intervention (three acute urinary retention and 2 urethral stricture).

A summary of included studies is shown in Table 1. Reports of included studies are available in the references [19,20,21,22,23,24,25,26,27,28,29,30,31,32,33,34,35,36,37,38,39,40,41,42,43,44,45,46,47,48,49,50,51].

The overall methodological quality of the included studies was variable.

The single randomized, controlled clinical trial [6] was assessed with the ROB-2 tool and demonstrated a low risk of bias across all domains, including randomization process, deviations from intended interventions, completeness of outcome data, measurement of outcomes, and selective reporting.

The prospective, multi-center comparative study by Quintas et al. [13], which evaluated Aquablation against HoLEP, was assessed using the ROBINS-I tool. The overall risk of bias was judged as serious, mainly due to concerns regarding confounding and selection into interventions. Treatment allocation was based on patient choice rather than randomization, raising the possibility of baseline differences between groups. In addition, surgeon experience varied across centers, potentially influencing safety outcomes. Other domains, including completeness of outcome data, measurement of outcomes, and selective reporting, were considered to be at low risk of bias.

Risk of bias assessment for the studies is shown in Table 2 and Table 3.

## 4. Discussion

This systematic review synthesizes evidence from randomized controlled trials and real-world studies evaluating the safety profile of Aquablation for the treatment of benign prostatic hyperplasia (BPH), with a particular focus on adverse events and complications. Across both controlled and observational cohorts, Aquablation consistently exhibits a favorable perioperative morbidity profile, with complication rates that are typically lower than or comparable to those associated with conventional surgical techniques, such as TURP. Both the American and European Association of Urology Guidelines suggest that Aquablation may be offered to patients with LUTS and prostate volume 30–80 mL with evidence level grade C and weak strength rating, respectively [52,53]. Most importantly, Aquablation has been shown to preserve sexual function, especially antegrade ejaculation, according to findings from multiple published studies. The improved ejaculatory outcomes after Aquablation are attributed to precise surgical mapping and tissue resection that avoids heat damage to key anatomical landmarks, such as the ejaculatory ducts.

An increasing number of studies reporting comparative outcomes with other treatment modalities are emerging. In a prospective, multi-center study by Quintas et al. (2025), Aquablation was directly compared with HoLEP [13]. Both procedures demonstrated comparable complication rates and transfusion requirements; however, Aquablation resulted in a greater peri-operative reduction in hemoglobin (2.6 g/dL vs. 0.4 g/dL). Notably, Aquablation demonstrated a significant advantage in preserving ejaculation, with only 6.6% anejaculation rate, compared to 89.3% in the HoLEP group, underscoring its distinctive functional benefit.

As postoperative complications are an unavoidable aspect of surgical outcomes, their frequency can be mitigated through growing surgical expertise, especially in high-volume centers. Bleeding is the most common peri-operative concern regarding Aquablation, especially for large prostates. Hemorrhagic events usually occur early after surgery, are manageable and rarely recur beyond 30 days postoperatively. Life-threatening complications are rare, and long-term problems, such as strictures or incontinence, are uncommon. Functional results, including preservation of ejaculation and erectile function, are consistently superior to TURP or HoLEP. Furthermore, it should be acknowledged that surgeon experience and the procedural learning curve may influence early complication rates. As with other new surgical technologies, outcomes tend to improve with increased familiarity and standardization of technique. Studies have shown that Aquablation performance stabilizes after a relatively short learning phase, particularly in high volume centers, which may partially explain the variation in perioperative bleeding or re-intervention rates observed across studies [15].

An emerging aspect of the evidence base concerns the influence of prostate size on perioperative morbidity. Data from larger prospective cohorts indicate that bleeding-related complications and transfusion requirements occur somewhat more frequently in prostates ≥150 mL, whereas smaller and medium-sized glands generally demonstrate low complication rates and favorable functional recovery. Despite the modest increase in bleeding risk with large glands, the overall incidence of major adverse events remains low, and CD grade ≥ 3 complications are uncommon. These findings imply that the precision and heat-free resection mechanism of Aquablation may attenuate the risks typically associated with surgery for large prostates, supporting its potential applicability across a wide range of prostate volumes.

The growing volume of independent data, coupled with stratified analyses according to prostate size, has refined the understanding of Aquablation’s safety profile. Collectively, the current body of evidence indicates that while perioperative bleeding remains the predominant concern, serious complications are rare, and functional outcomes, particularly preservation of ejaculation and continence, remain consistently favorable across patient populations and study settings. As experience with the technology broadens globally, these findings underscore its reliability and reproducibility in diverse clinical contexts.

Recent literature has highlighted several factors influencing the safety and clinical outcomes of Aquablation, extending beyond the early findings of pivotal trials. Evidence published over the past few years has expanded the understanding of its performance across different clinical environments, encompassing both industry-sponsored and independent investigations. Early multicenter trials such as WATER and WATER II, which were largely manufacturer-supported, established the initial safety profile of the procedure. More recently, independently conducted studies from Europe, North America, and Asia have reported comparable perioperative and functional outcomes, suggesting that the favorable safety characteristics of Aquablation are reproducible in real-world practice. The consistency between sponsored and independent datasets enhances the credibility of the available evidence and supports the generalizability of the reported safety profile.

When positioned within the broader landscape of minimally invasive surgical therapies (MISTs) for BPH, Aquablation demonstrates a distinctive profile in balancing efficacy, safety, and preservation of sexual function. Modalities such as Rezūm, UroLift and iTind are effective alternatives for selected patients, particularly those with smaller prostate volumes (<80 mL), and are characterized by minimal perioperative morbidity and excellent preservation of ejaculation. However, these approaches are generally associated with higher retreatment rates and less pronounced improvements in symptom scores compared with resection-based procedures. In contrast, Aquablation achieves symptom relief and functional improvement comparable to TURP and HoLEP, while maintaining the low morbidity profile typical of MISTs. As discussed in recent comparative analyses [54], Aquablation extends the applicability of minimally invasive therapy to larger prostates, bridging the gap between non-ablative office-based interventions and traditional endoscopic resection. This positions Aquablation as an intermediate option that offers durable outcomes with preservation of sexual function across a wide spectrum of prostate sizes.

Despite these advantages, Aquablation also presents certain constraints when compared with other minimally invasive options. The requirement for general or spinal anesthesia, specialized robotic equipment, and operative settings restricts its availability to tertiary centers and increases procedural costs relative to office-based MISTs such as Rezūm or UroLift. Operative time and the potential for perioperative bleeding also remain higher than with non-resective techniques. Furthermore, while functional preservation and symptom improvement appear durable, long-term comparative data beyond five years are still limited. Continued evaluation through independent, randomized studies directly comparing Aquablation with other MISTs will be essential to define its optimal role within the contemporary management of BPH.

From an economic and resource perspective, the adoption of Aquablation is influenced by the high upfront cost of the robotic platform and disposable handpieces, as well as the need for specialized equipment and trained personnel. Although the procedure may offer reduced hospital stay and lower retreatment rates, which could offset some expenses over time, the overall cost-effectiveness remains to be clearly established. Furthermore, access to Aquablation systems may currently be limited to tertiary or high-volume centers, particularly in regions where health system resources are constrained.

Assessment of methodological quality revealed notable variation between comparative designs. The randomized controlled trial by Gilling et al. [6] demonstrated a low risk of bias across all ROB-2 domains, indicating that its findings on perioperative safety and functional outcomes can be considered robust and internally valid. This supports confidence in the comparative evidence showing lower rates of ejaculatory dysfunction and similar overall complication frequencies between Aquablation and TURP. In contrast, the non-randomized comparative study by Quintas et al. [13] was judged to carry a serious overall risk of bias under ROBINS-I, primarily due to potential confounding and the absence of random allocation. Treatment choice in that study was based on patient preference and surgeon discretion, introducing a likelihood of selection bias. Although outcome measurement and reporting were methodologically sound, the lack of randomization limits the strength of causal inference when comparing Aquablation with HoLEP. As such, while the results of that study are valuable for hypothesis generation and for understanding real-world practice patterns, they cannot be interpreted as confirmatory evidence of equivalence or superiority between techniques. Taken together, the risk-of-bias assessment indicates that the current comparative evidence on Aquablation safety is driven largely by a single, low-bias randomized trial, supplemented by non-randomized data of lower methodological rigor. This underscores the need for further independent randomized studies to validate comparative findings and to establish the long-term safety profile of Aquablation with higher certainty.

This systematic review has several limitations that should be acknowledged. There was significant variability among the studies, including differences in design, inclusion criteria, follow-up periods, and adverse event definitions. While some used standardized frameworks, like Clavien-Dindo, others only reported certain complications, making direct comparison difficult. Follow-up duration also varied widely, from short-term (three months) to longer-term, with series extending to five years, raising the possibility that late complications, such as urethral stricture, bladder neck contracture, or need for re-treatment may be under-reported in many cohorts. Moreover, many real-world studies were relatively small, often including 50–60 patients, limiting the ability to detect rare but clinically important complications. Geographic representation is also uneven, with most trials conducted in North America and Europe, and only limited Asian experience is reported, raising questions about global generalizability. Finally, publication bias cannot be excluded, as the majority of studies to date have been industry-supported or performed in centers of excellence, which may underrepresent less favorable outcomes in routine practice. Taken together, these factors indicate that, although the available evidence consistently supports the safety of Aquablation, particularly regarding preservation of sexual function, conclusions should be interpreted with caution, until more independent, long-term, comparative trials are available.

## 5. Conclusions

This systematic review demonstrates that Aquablation tends to be a safe and effective surgical option for men with benign prostatic hyperplasia across a wide range of prostate volumes, including glands traditionally managed with open or enucleative procedures. Real-world evidence from diverse international cohorts supports the reproducibility of these outcomes outside controlled trial settings. While longer-term, independent, comparative data are still needed, the current literature indicates that Aquablation offers a favorable balance between efficacy, safety, and functional preservation, representing a valuable addition to the armamentarium of BPH surgical therapy.

## Figures and Tables

**Table 1:** Summary of studies included in the review.

Author	Year	Design	Single- or Multi-Centered	Population	Use of Clavien-Dindo Classification
Bach et al. [15]	2019	prospective, observational	single	118	yes
Bach et al. [10]	2020	prospective, single-arm clinical trial (OPEN WATER)	multi	178	yes
Desai et al. [18]	2018	prospective, single-arm clinical trial (AQUABEAM India Study)	single	47	yes
Desai et al. [9]	2019	prospective, single-arm clinical trial (WATER II)	multi	101	yes
Gilling et al. [6]	2018	randomized, controlled clinical trial (WATER)	multi	117 vs. 67 (TURP)	yes
Hinata et al. [14]	2025	prospective, observational	single	103	yes
Kasraeian et al. [17]	2020	prospective, observational	single	55	no
Labban et al. [16]	2021	prospective, observational	single	59	yes
Omidele et al. [11]	2024	prospective, observational	single	330	no
Quintas et al. [13]	2025	prospective, comparative	multi	75 vs. 75 (HoLEP)	yes
Ringler et al. [12]	2025	prospective, observational	single	174	yes

**Table 2:** Risk of bias assessment for randomized, controlled clinical trial.

Study	Randomization Process	Deviations from Intended Interventions	Missing Outcome Data	Measurement of the Outcome	Selection of the Reported Result	Overall Risk of Bias
Gilling et al. 2018 [6]	Low	Low	Low	Low	Low	Low

**Table 3:** Risk of bias assessment for non-randomized clinical trial.

Study	Confounding	Selection of Participants	Classification of Interventions	Deviations from Intended Interventions	Missing Data	Measurement of Outcomes	Selection of Reported Result	Overall
Quintas et al. 2025 [13]	Serious	Serious	Low	Moderate	Low	Moderate	Low	Serious

## Data Availability

The raw data supporting the conclusions of this article will be made available by the authors on request.

## Abbreviations

The following abbreviations are used in this manuscript:

BPH	Benign Prostatic Hyperplasia
CD	Clavien-Dindo
HoLEP	Holmium Laser Enucleation of the Prostate
iTIND	Temporary Implantable Nitinol Device
LUTS	Lower Urinary Tract Symptoms
MIST	Minimally Invasive Surgical Therapy
TURP	Transurethral Resection of the Prostate
Rezūm	Convective Water Vapor Thermal Therapy
UroLift	Prostatic Urethral Lift
